# Subtenon injection of natural leukocyte interferon α-2a in diabetic macular edema: a case report

**DOI:** 10.1186/1471-2415-13-63

**Published:** 2013-10-28

**Authors:** Mauro Cellini, Nicole Balducci, Ernesto Strobbe, Emilio C Campos

**Affiliations:** 1 Department of Specialized, Diagnostic and Experimental Medicine, Ophthalmology Unit, University of Bologna, S. Orsola Malpighi-Hospital, Pelagio Palagi 9, Bologna 40138, Italy

**Keywords:** Diabetic macular edema, Interferon, Subtenon injections, Central macular thickness, Spectral domain optical coherence tomography

## Abstract

**Background:**

To report the effect of subtenon injections of natural leukocyte interferon α-2a (IFNα) on best corrected visual acuity (BCVA) and central macular thickness (CMT) in a patient with diabetic macular edema (DME).

**Case presentation:**

A 66-year-old man affected by DME, with glycated hemoglobin (HbA1c) at 6.9%, refractory to laser grid treatment and intravitreal injections of triamcinolone, was selected to receive a cycle of three subtenon injections/week of IFNα (1×10^6^ IU/ml). BCVA and CMT, using spectral domain ocular coherence tomography (SD-OCT), were evaluated preoperatively and at 1 week, 1 month, 4 months, and 1 year postoperatively. BCVA and CMT were significantly improved at 1 week after the three injections (20/200 *vs.* 20/40 and 498 μm *vs.* 237 μm, respectively). BCVA remained stable during the 1-year follow-up. CMT was slightly increased, but was still lower than the baseline value (215 μm, 255 μm, and 299 μm during the follow-up visits). No adverse events were recorded, with the exception of mild subconjunctival hemorrhage at the injection site.

**Conclusions:**

IFNα, with its immunomodulatory, anti-proliferative and anti-angiogenic actions, was effective in improving BCVA and reducing CMT in refractory DME. Further randomized controlled studies are required to assess the effect of IFNα alone or in combination with other therapies for DME treatment.

## Background

Diabetes mellitus (DM) is a common disease and the macular edema it causes is the leading cause of visual loss around the world. Diabetic macular edema (DME) is due to the breakdown of the blood-retinal barrier secondary to complex biochemical dysregulation [1].

Despite focal and/or grid argon laser photocoagulation, which is considered the standard of care in clinically significant DME, a distinct subgroup of eyes with DME is resistant to conventional laser therapy, with visual acuity continuing to decline [2].

More recently, the role of oxidative stress, inflammatory mediators (like interleukin [IL]-6, IL-8, monocyte chemotactic protein-1, tumor necrosis factor [TNF-α], protein kinase [CPKC-β], nitric oxide synthase, nuclear factor kappa-light-chain-enhancer of activated B cells [NF-κB]) and the up-regulation of vascular endothelial growth factor (VEGF) in DME have been elucidated [1]. Thus, intravitreal (IV) injections of corticosteroids [3] and/or anti-VEGF drugs [4] have been proposed alone or in combination to treat DME.

However, these treatments have drawbacks. For example, IV injection of corticosteroids shows short-term effects and high rates of intraocular complications [3], whereas anti-angiogenic drugs provide a definite, but small, benefit compared to current therapeutic options for DME (i.e., grid laser photocoagulation), requiring also a large number of injections and high costs [4].

Interferons belong to a large class of glycoproteins known as cytokines and are released by host cells in response to the presence of pathogens or tumor cells. Interferons have anti-viral, immunomodulatory, and anti-proliferative properties, inhibiting VEGF and other cytokines like IL-8, IL-10, tumor growth factor [TGF-β], and TNF-α [5], while enhancing the barrier function of the retinal microvascular endothelium *in vitro*[6].

In some cases, we have used these anti-proliferative and anti-VEGF properties, with an off-label subtenon injection of IFNα in patients with diabetic macular edema [7] and age-related choroidal neovascularization [8] and we have followed them for a period of 4 months reporting good results.

This case report highlights, for the first time, the effect of subtenon injections of natural leukocyte interferon α-2a (IFNα) on best corrected visual acuity (BCVA) and central macular thickness (CMT) in a patient affected by refractory DME after one year that the treatment was stopped.

## Case presentation

A 66-year-old man, affected by type II DM for 4 years and arterial hypertension for 10 years, developed severe non-proliferative diabetic retinopathy without macular edema just 2 years after DM diagnosis. The patient was treated with panretinal photocoagulation (PRP), because he had a poor glycometabolic control and was unable to adhere to a close follow-up. One year after PRP, he developed severe visual loss due to diffuse non tractional bilateral DME. Thus, the two eyes were treated with an IV triamcinolone injection 1 month apart (the right eye [RE] before the left eye [LE]) and laser grid photocoagulation was performed in both eyes 2 weeks after IV injection. After no visual recovery for over 2 months due to the persistence of macular edema in both eyes, an IV injection of bevacizumab was given in the RE, where a larger amount of intraretinal fluid and subfoveal neural detachment were observed. However, 1 week after the injection, anterior ischemic optic neuropathy (AION) with a residual visual acuity of hand motion was observed. During the entire period, the patient had poor glycometabolic control (glycated hemoglobin >9%, high hypertension, and hypercholesterolemia) and poor adherence to therapy. This fact could have influenced the patient’s poor response to therapy and the development of AION.

As a result of this unfortunate adverse event, the patient refused an IV injection of bevacizumab in his LE. After informing the patient about the risks and benefits of different treatment options such as observation, off-label IV injections of steroids, and subtenon injections of IFNα, the patient signed an informed consent for a cycle of subtenon injections of IFNα.

Before the treatment, the BCVA in the LE was 20/200. The patient received posterior subtenon injections of IFNα (1×10^6^ IU/ml) three times (on Monday, Wednesday, and Friday) for a week in his LE, according to the following procedure: after the administration of topical 0.4% oxybuprocaine surface anesthesia, 1 ml of IFNα was slowly injected into the inferotemporal quadrant under the Tenon’s capsule, using a 27-gauge needle on a 2.5-ml syringe. The needle was moved toward the macular area, until the hub was firmly pressed against the conjunctival fornix. After the first injection, topical 0.3% netilmicin eye drops were prescribed three times a day for 7 days. During the period of IFN-α therapy, the patient had a good systemic condition and a good glycometabolic control (glycated hemoglobin = 6.9%).

**Figure 1:**
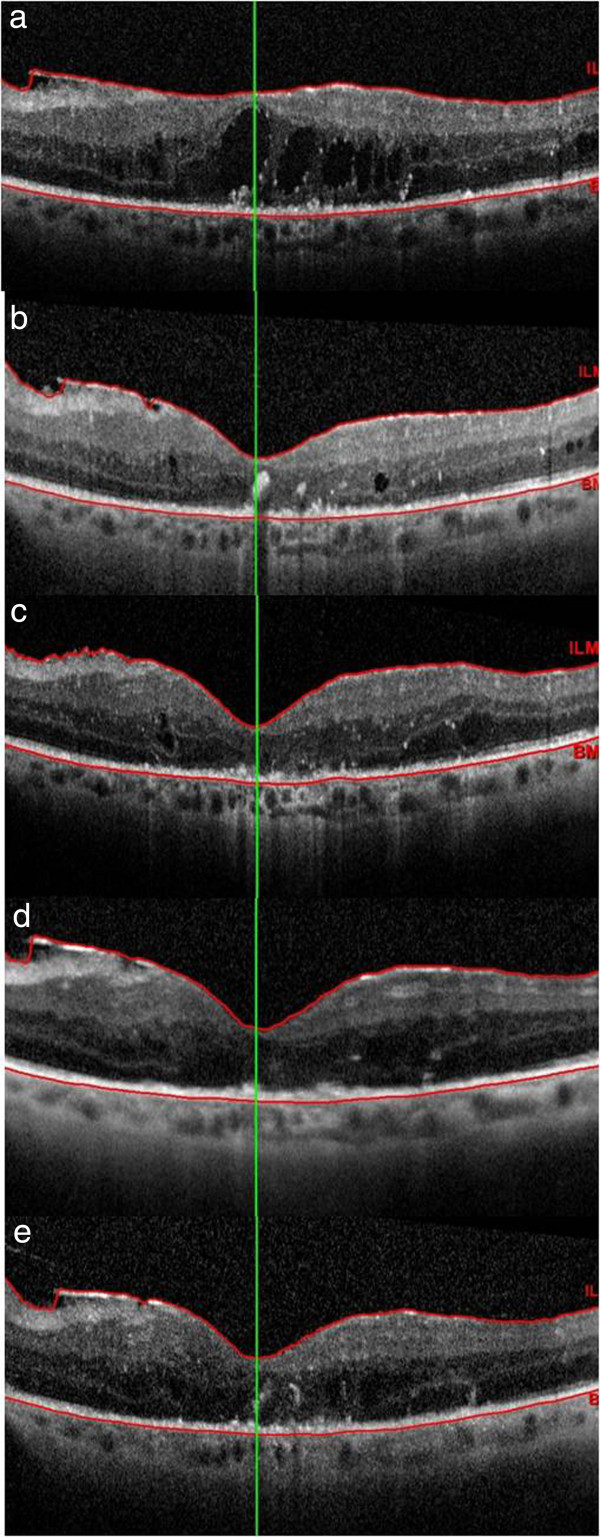
**Central macular thickness (CMT) modification during the follow-up visits measured with spectral domain optical coherence tomography.** At baseline, CMT was 497 μm **(a)**; one week after 3 posterior subtenon injections of natural leucocytic interferon-α 2a. CMT decreased to 237 μm **(b)**; after 1 month **(c)**, 3 months **(d)** and 1 year **(e)** CMT was 215, 255 and 299 μm, respectively.

**Figure 2:**
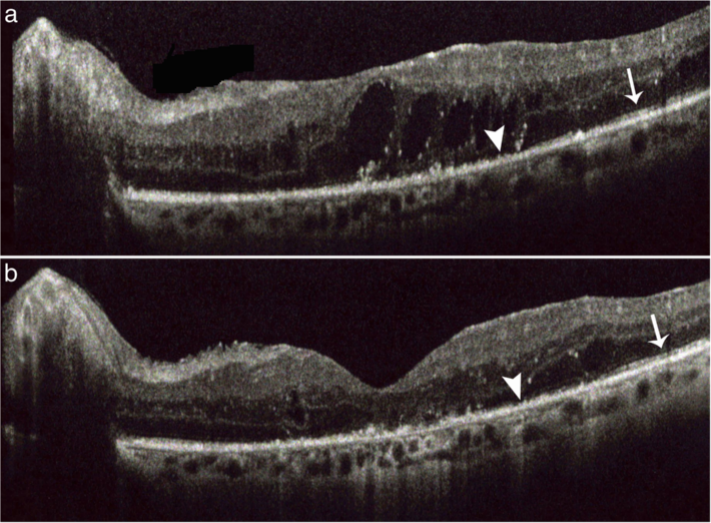
**Photoreceptor inner segment / outer segment (IS / OS) at the baseline and after 15 months of follow-up.** The figure shows the focal presence (arrow) and the focal central disruption (arrow head) of the IS/OS layer before the IFN-α injections **(a)**. Fifteen months after treatment we found a decrease of focal central disruption (arrow head) of the IS/OS layer **(b)**.

A complete ophthalmic examination including BCVA, indirect ophthalmoscopy, and SD-OCT of the macular region was conducted several times throughout the period of IV injection and laser grid therapy and, specifically, preoperatively, as well as at 1 week, 1 month, 4 months, and 1 year after the last injection of INFα. SD-OCT images were obtained using Spectralis OCT spectral-domain (Heidelberg Engineering GmbH, Heidelberg, Germany) and the baseline macular scan was set as the reference (Figure 1). Fifteen months after the treatment, the BCVA in the LE was 20/40, with reduced cystoid macular edema but the SD-OCT highlighted the persistence of the photoreceptor inner segment/outer segment (IS/OS) disruption already highlighted at the baseline (Figure 2). The patient gives his written consent for the use of his data and any accompanying images.

## Conclusion

This case report demonstrates that IFNα may be an effective therapeutic option for refractory DME in patients who show contraindications or complications from IV anti-VEGF injections. Indeed, it may improve BCVA and CMT by acting against different biochemical targets involved in the pathogenesis of DME, such as VEGF, IL-8, IL-10, TGF-β, and TNF-α [5,6], even if its effect decreases slightly over time. The disruption of the IS/OS may be related not only to the period of about 1 year between the onset of macular edema and the resorption of intraretinal fluid [9] after IFN therapy, but also to an apoptotic action of the IFN, with the formation of capsase-4 and -8 as already noted with other anti-VEGF agents [10].

Due to its anti-inflammatory and anti-proliferative properties, intralesional injection of IFN has been used to treat conjunctival lymphoma [11,12], while systemic IFN has been applied to treat non-infectious uveitic chronic macular edema [13]. Within 2 to 4 weeks, approximately 94% of patients were seen to reach a complete or partial remission in the case of Behҫet disease-associated uveitis. Moreover, IFNα is the only drug that leads to stable remission even after the discontinuation of treatment [13]. A pilot study provided evidence that systemic IFN may have a key role in the regression of proliferative diabetic retinopathy [14].

Although IFNα is a large protein molecule, we decided to administer it by subtenon injection because it can penetrate the sclera and enter the choroid, as demonstrated by Lincoff [15]. In fact, after retrobulbar injection, IFNα reaches higher intravitreal concentrations than systemic injection and is well tolerated because a lower dosage is required [15]. None of the adverse events described in the literature after systemic IFN administration, such as flu-like symptoms, leucopenia, or depression [13], were found in our patient.

This case report provides evidence that IFNα might have a role in the treatment of DME. However, further randomized controlled studies are required to assess the effect of IFNα alone or in combination with other therapies for DME treatment.

## Competing interests

The authors declare that they have no competing interests.

## Authors’ contributions

MC recruited the patient from the Ophthalmology First Aid of the S. Orsola-Malpighi Hospital and performed the injection; NB and ES drafted the manuscript and reviewed the literature; ES and ECC evaluated OCT. All authors read and approved the final manuscript.

## Pre-publication history

The pre-publication history for this paper can be accessed here:

http://www.biomedcentral.com/1471-2415/13/63/prepub
